# Unveiling the role of interleukin-6 in pancreatic cancer occurrence and progression

**DOI:** 10.3389/fendo.2024.1408312

**Published:** 2024-05-17

**Authors:** Meihui Song, Ying Tang, Kaimei Cao, Ling Qi, Keping Xie

**Affiliations:** ^1^ School of Medicine, South China University of Technology, Guangzhou, Guangdong, China; ^2^ Division of Gastroenterology, Institute of Digestive Disease, Qingyuan People’s Hospital, The Affiliated Qingyuan Hospital of Guangzhou Medical University, Qingyuan, Guangdong, China; ^3^ Department of Gastroenterology, The Third Affiliated Hospital of Sun Yat-Sen University, Guangzhou, Guangdong, China; ^4^ School of Pharmaceutical Sciences, Dali University, Dali, Yunnan, China

**Keywords:** IL-6, pancreatic cancer, PanIN, tumor immunity, cachexia, diagnostic biomarker

## Abstract

Pancreatic cancer is difficult to diagnose early and progresses rapidly. Researchers have found that a cytokine called Interleukin-6 (IL-6) is involved in the entire course of pancreatic cancer, promoting its occurrence and development. From the earliest stages of pancreatic intraepithelial neoplasia to the invasion and metastasis of pancreatic cancer cells and the appearance of tumor cachexia, IL-6 drives oncogenic signal transduction pathways and immune escape that accelerate disease progression. IL-6 is considered a biomarker for pancreatic cancer diagnosis and prognosis, as well as a potential target for treatment. IL-6 antibodies are currently being explored as a hot topic in oncology. This article aims to systematically explain how IL-6 induces the deterioration of normal pancreatic cells, with the goal of finding a breakthrough in pancreatic cancer diagnosis and treatment.

## 1 Introduction

IL-6 is a glycoprotein, initially known as B cell stimulating factor 2 due to its capability to induce B cells to produce immunoglobulin upon its early discovery (1, 2). Upon further study of this factor, it was revealed that its functions extend beyond immune regulation to encompass areas such as inflammation, hematopoiesis, and tumors, prompting its renaming as IL-6. Within healthy individuals, IL-6 is produced by a range of normal cells including hematopoietic cells, monocytes, macrophages, epithelial cells, and muscle cells, contributing to neural development, immune response, and angiogenesis (3). IL-6 is promptly and transiently generated in reaction to infections and tissue damage, aiding host defense by triggering acute phase responses, promoting hematopoiesis, and enhancing immune reactions. While its production is tightly regulated by, persistent dysregulated synthesis of IL-6 can lead to pathological effects such as chronic inflammation and autoimmunity (4).

Pancreatic cancer stands out as the most malignant tumor within the digestive system and is a leading cause of cancer-related deaths globally. This disease often progresses without early symptoms, leading to late-stage detection and a poor prognosis (5, 6). The tumor microenvironment (TME) significantly influences tumor progression, with inflammatory cells releasing tumor-promoting cytokines. Prolonged exposure to these cytokines can lead to chronic pancreatitis, contributing to pancreatic cancer development and progression. With the increasing incidence and mortality rates of pancreatic cancer, there is a growing emphasis on identifying effective diagnostic and treatment approaches. Recent studies suggest that biotherapy holds promise as a crucial breakthrough in managing pancreatic tumors, garnering considerable attention from clinicians and researchers (7).

Biotherapy involves identifying targets at the immune system and molecular level, which relies on cytokines to facilitate cellular communication. Research has demonstrated that IL-6, a multifunctional cytokine, is overexpressed in pancreatic cancer (8). The signal transduction facilitated by IL-6 plays a vital role in mediating interactions between tumor cells and stromal cells. This is particularly significant in shaping the microenvironment of pancreatic cancer, providing conducive conditions for cancer progression and metastasis (9). In this paper, we aim to comprehensively expound upon the mechanisms through which IL-6 contributes to the onset and progression of pancreatic cancer, while also highlighting potential directions for clinical treatment of the disease.

## 2 IL-6 is involved in pancreatic intraepithelial neoplasia

Pancreatic intraepithelial neoplasia (PanIN) is the most common precursor lesion of pancreatic cancer (10), often resulting from Kras protein mutation. In cases where the Kras protein remains unmutated, it can facilitate tissue repair when pancreatic lesions arise, promoting the swift restoration of normal cell morphology and pancreatic function. Conversely, the mutated Kras protein transforms into an oncogene, functioning as a molecular switch that transmits signals downstream through two pathways. One involves the RAF/MEK/ERK signal pathway within the MAPK family, while the other entails the PI3K/AKT signal pathway. Ultimately, the phosphorylated substrate triggers the onset of pancreatic cancer (11, 12). However, simply relying on the Kras gene is insufficient to drive pancreatic carcinogenesis. Recent studies suggesting that the role of IL-6 in PanIN acts synergistically with Kras protein to promote pancreatic carcinogenesis have generated considerable debate and discussion.

In a genetically engineered mouse model of pancreatic cancer known as the iKras* mouse (p48-Cre; R26-rtTa IRES-EGFP; TetO-KrasG12D), characterized by pancreas-specific, inducible, and reversible expression of oncogenic KrasG12D (Kras*), the pancreas exhibited significantly elevated levels of IL-6 mRNA expression compared to the wild control group. Interestingly, a decrease in PanIN formation was observed in IL-6 deficient iKras* mice, indicating a clear link between IL-6 and PanIN. The absence of IL-6 led to reduced levels of MAPK and PI3K/Akt signaling, which correlated with a decrease in PanIN formation. This suggests that IL-6 is essential for the downstream signaling of the Kras gene (13–15).

PanIN relies on IL-6 to trigger the reactive oxygen species (ROS) detoxification program. Accumulation of ROS poses a major threat to tumor cells, therefore, scavenging ROS is a critical step in tumorigenesis (16). Despite mutant Kras playing a pivotal role in regulating redox homeostasis (17), IL-6 remains essential for bolstering the antioxidant stress capacity of tumor cells. IL-6 increases the expression of Nrf2, a crucial transcription factor that facilitates the ROS detoxification program (13). IL-6 and Kras genes collaborate to advance the progression of PanIN to pancreatic cancer, with the signals they generate playing a crucial role in tumor growth.

## 3 The signal transduction of IL-6 contributes to the progression of pancreatic cancer

The metabolic rate of cancer cells surpasses that of normal cells, resulting in a frequently hypoxic environment. To ensure survival, the upregulation of the IL-6/STAT3 pathway triggers the synthesis of vascular endothelial growth factor (VEGF) and promotes the proliferation of tumor blood vessels, creating favorable conditions to meet the heightened nutritional demands of tumor cell (18). Furthermore, with the overactivation of the STAT3 protein through the IL-6 signaling pathway, there is an increase in the expression of cyclin D1 and Bcl-2 genes (19).

**Figure 1:**
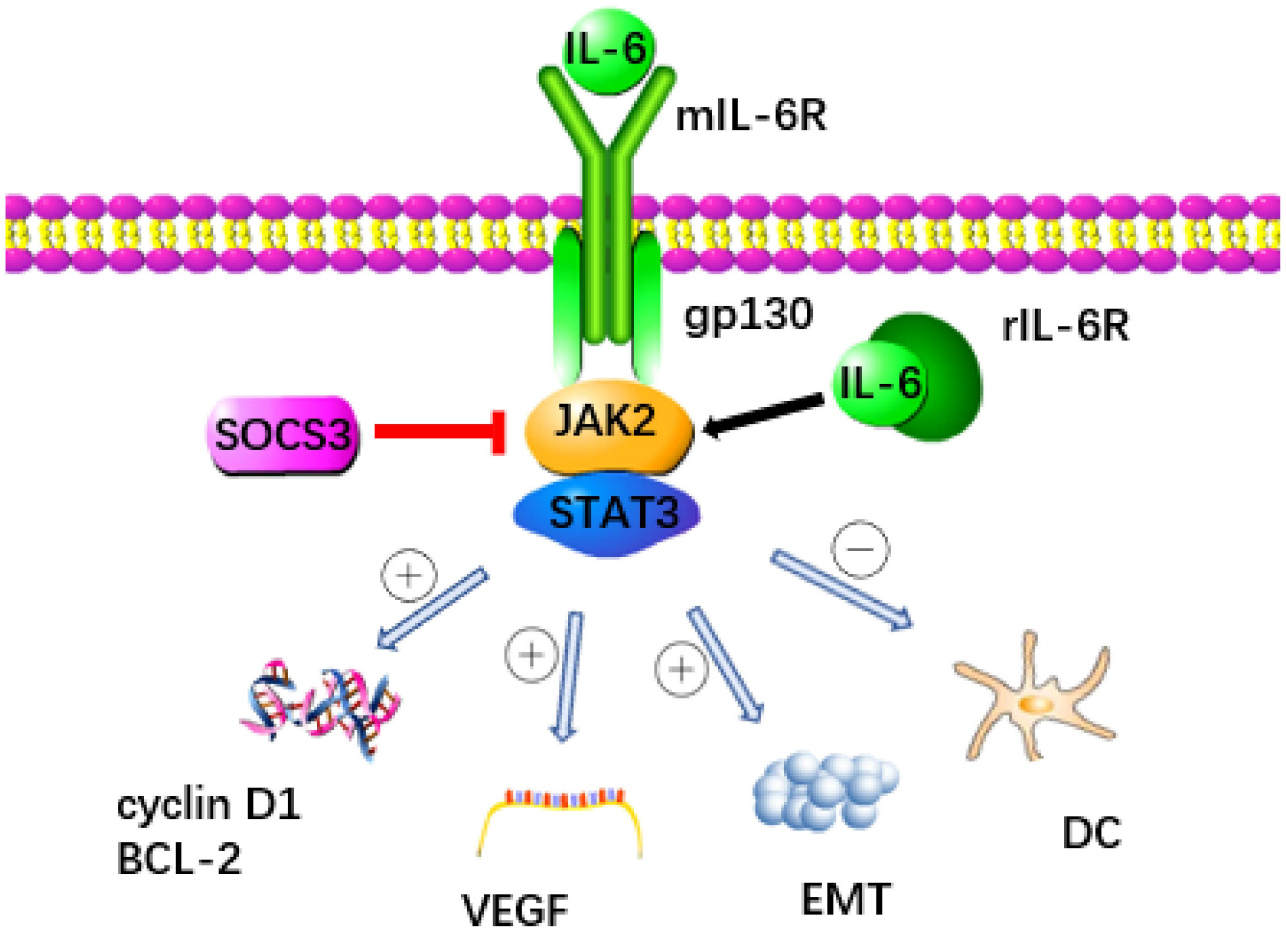
The signaling pathway triggered by IL-6 is recognized as a key contributor to the development of pancreatic cancer. IL-6 binds to mIL-6R or rIL-6R to activate JAK2 and transmit signal to downstream STAT3 protein. This process was inhibited by SOCS3. STAT3 promotes the progression of pancreatic cancer through a variety of ways: (1) Promoting the expression of cyclin D1 and Bcl-2 genes, (2) Up regulating VEGF, (3) Accelerating the occurrence of EMT, (4) Inhibiting the immune function of DC.

The IL-6 signaling pathway is a key factor in the development of pancreatic cancer, with the IL-6/JAK/STAT pathway playing a particularly crucial role. This pathway facilitates signal transduction through two distinct mechanisms (**Figure 1**) (20, 21). When IL-6 binds to the membrane-bound IL-6 receptor (mIL-6R) to activate STAT protein, it’s known as classical signal transduction (22, 23). In contrast, when IL-6 binds to soluble IL-6R (sIL-6R), resulting from the cleavage and shedding of mIL-6R, and transmits signals downstream, it’s termed trans signal transduction (24, 25). IL-6 can bind mIL-6R or sIL-6R, forming a dimer and initiating activation of transmembrane gp130, leading to the association of the box region in gp130 with JAK2 to trigger the activation of the STAT3 protein (15, 26).

Cytokine signal transduction inhibitor 3 (SOCS3) functions as a crucial negative regulator within the IL-6/JAK2/STAT3 pathway, effectively curbing STAT3 overactivation and maintaining equilibrium (19). SOCS3 not only directly decreases the catalytic activity of JAKs but also enhances the ubiquitination of gp130 and JAK2 by increasing components of an E3 ubiquitin ligase complex, thereby impeding downstream signal transmission from IL-6 (27, 28). Moreover, the E3 ubiquitin ligase NEDD4L interacts with GP130, promoting its ubiquitination in tumors and thwarting the activation of the IL-6/GP130/STAT3 signaling axis, thereby exhibiting anti-cancer properties (29). Various members of the membrane-bound E3 ubiquitin ligase family, including MARCH2, MARCH3, MARCH4, and MARCH9, target cell surface receptors for degradation, leading to a reduction in IL-6 responsiveness, primarily through a decrease in cell surface expression levels of IL-6R (30). Recent research has pinpointed C1q/TNF-related protein 4 (CTRP4) as a natural regulator of the IL-6 receptor signaling pathway. CTRP4 competes with IL-6 for binding to IL-6R, thereby dampening IL-6-induced activation of the STAT3 pathway (31). These negative regulatory pathways governing IL6 activity are pivotal in preventing excessive responses to infections and maintaining endogenous IL6 levels. Manipulating these pathways holds promise for therapeutic interventions in tumors, as well as a broad spectrum of human autoimmune and inflammatory conditions (32).

## 4 Cancer cell metastasis is closely linked to IL-6

### 4.1 IL-6 enhances immune evasion by influencing Th2 cells

Cancer cells frequently evade attacks from the immune system and encroach upon the territory of normal cells, particularly in the case of pancreatic cancer. This immune evasion is attributed to issues within various immune regulatory pathways, including an imbalance in the Th1/Th2 ratio of helper T cells (33). Both Th1 and Th2 cells originate from pre-CD4+T cells, and the differentiation into each type of cell is largely influenced by the cytokine environment (34). Th1 cells serve as the primary force of the immune response, rallying other cells (macrophages, NK cells, and T cells) to ward off foreign cell invasion. On the other hand, Th2 cells are generated from CD4+T cells under the influence of Th2 cytokines (such as IL-4, IL-5, IL-10, and IL-13). In contrast to the function of Th1 cells, Th2 cells can suppress the combat effectiveness of immune cells and protect their own cells from destruction (35). In normal conditions, Th1 and Th2 cells exert inhibitory effects on each other’s proliferation. Maintaining a balance between them is crucial for preserving overall bodily health. Any disruption in this balance, leading to a biased shift toward one side (referred to as “Th1/Th2 drift”), can contribute to the onset of disease (36).

Elena discovered that CD4+T cells in the serum of pancreatic cancer patients exhibited increased production of the Th2 cytokine IL-5, resulting in a phenotype of tumor-infiltrating lymphocytes that favored Th2 cells (37). An excessive presence of Th2 cells may result in the suppression of Th1 cells, indicating that an imbalance in Th1/Th2 cell levels could potentially play a role in the onset and progression of pancreatic cancer (38, 39). Hence, understanding the cause of the upregulation of Th2 cells is a potential avenue to improve outcomes for pancreatic cancer patients. Louis W and his colleagues investigated the cytokine environment surrounding Th2 cells and observed a significant increase in IL-13 and IL-10 secretion induced by IL-6. Their findings suggest that IL-6 can stimulate pancreatic cancer cells to release Th2 cytokines, consequently fostering the generation of Th2 cells and indirectly facilitating immune evasion by tumor cells (40). Furthermore, there is evidence indicating that IL-6 can enhance the expression of the Th2 cytokine IL-4 gene by activating NFAT transcription factors, thereby facilitating the differentiation of T cells into Th2 cells (41). It is important to counteract the influence of IL-6 on helper T cells in order to preserve the balance of Th1/Th2 and bolster anti-tumor immunity.

### 4.2 The metabolic reprogramming influenced by IL-6 impacts immune cells

Altered energy metabolism is a characteristic of pancreatic cancer. In order to fulfill the growth requirements of tumor cells, there is an observed increase in glycolytic flux. When blood glucose levels are low or cellular carbohydrate reserves (e.g., glycogen) are depleted by malignant cells, liver cells metabolize fatty acids into ketones to generate additional energy and maintain the normal function of tissue cells. This self-protective response is known as the action of ketone bodies. It has been suggested that the metabolic reprogramming induced by ketone bodies may mitigate the cachexia associated with pancreatic cancer (42). However, patients with pancreatic cancer experience a reduced capacity for ketogenesis.

Flint et al. discovered that in cachexia mice, IL-6 diminished ketogenesis in the liver by inhibiting PPAR α (43). PPAR α plays a crucial role in activating ketogenic genes and serves as an essential transcription factor to initiate the process of ketogenic transcription (44). When IL-6 downregulates PPAR α, it restricts the ketogenic effect, leading to greater energy deficiency in cancer patients. Interestingly, this energy deficit can result in systemic metabolic stress, characterized by a notable increase in glucocorticoid levels. While synthetic glucocorticoids (such as dexamethasone) can be employed as apoptosis-inducing agents in clinical cancer treatment, endogenous glucocorticoids typically promote cancer progression (45). In a mouse model of pancreatic cancer, the injection of recombinant IL-6 not only elevated corticosterone levels but also led to the depletion of immune cells such as CD4+T cells, CD8+T cells, and NK cells (43). This evidence demonstrates that the hormone dysfunction induced by IL-6-driven metabolic reprogramming further influences anti-tumor immunity. It is conceivable that pancreatic cancer cells alter the host’s metabolism by secreting IL-6, and subsequently leverage the influence of hormones to bolster their defense. This positive feedback cycle is likely to result in the failure of immunotherapy (46).

## 5 IL-6 accelerates the occurrence of cachexia in pancreatic cancer

Cancer-related cachexia is present in over 85% of individuals with pancreatic cancer, significantly contributing to the low survival rates among these patients (47). In 2011, an international consensus defined cachexia as a wasting syndrome marked by progressive depletion of skeletal muscle mass (33), his may be accompanied by severe anorexia, significant weight loss, muscle weakness, cognitive decline, and other detrimental manifestations (48). Nevertheless, the impact of cachexia extends beyond muscle loss, affecting nearly all tissues and organs in the body (49). Tian and colleagues noted myocardial fibrosis and myocardial atrophy in a mouse model of cancer cachexia, indicating that cachexia may diminish cardiac contractility (50). Rosa discovered that cachexia triggered collagen accumulation in the liver, resulting in disrupted liver metabolism (51). Additionally, dysfunctions of the reproductive system, imbalances in intestinal flora, and endocrine changes in the islets are also associated with cachexia (49, 52). Consequently, researchers have started redirecting their focus toward the mediators of cancer cachexia in order to prevent these outcomes.

In recent years, cachexia has been found to be intricately linked to the host itself and numerous tumor-derived cytokines (53), with IL-6 being identified as one of the factors related to cachexia in pancreatic cancer. Clinical data statistics indicate that progressive weight loss, fatigue, and other cachexia symptoms in patients with advanced pancreatic cancer are often positively associated with serum IL-6 levels (54). Martinoni and colleagues conducted immunohistochemical staining on tissue sections from pancreatic cancer patients, they found that IL-6 exhibited greater immunoreactivity in tissue regions of patients with cachexia compared to those without cachexia (55). Furthermore, the administration of anti-IL-6 monoclonal antibody in mice was found to partially inhibit the progression of cancer cachexia (56). This indicates that IL-6 contributes to the development of cachexia in pancreatic cancer.

Cachexia primarily arises from the imbalance between catabolic and anabolic signals. Notably, IL-6 is implicated in both of these signaling pathways. During the early stages of cachexia, prior to skeletal muscle atrophy, fat depletion occurs as white adipose tissue (WAT) transforms into brown adipose tissue (BAT). Unlike WAT, BAT efficiently breaks down triglycerides stored in adipocytes into free fatty acids and glycerol, leading to impaired energy storage over time (57, 58). The process of white adipose tissue browning is viewed as the foundation of cachexia, and it is actually triggered by IL-6 (59, 60). Elevated levels of IL-6 can enhance the expression of uncoupling protein 1 (UCP1) (57), by disrupting the link between electron transport and phosphorylation in a segment of the typical respiratory chain, resulting in halted ATP synthesis. An anti-IL-6 receptor antibody was employed in treating cachexia in mice, this led to the inhibition of white adipose tissue browning and lipolysis (60).

**Figure 2:**
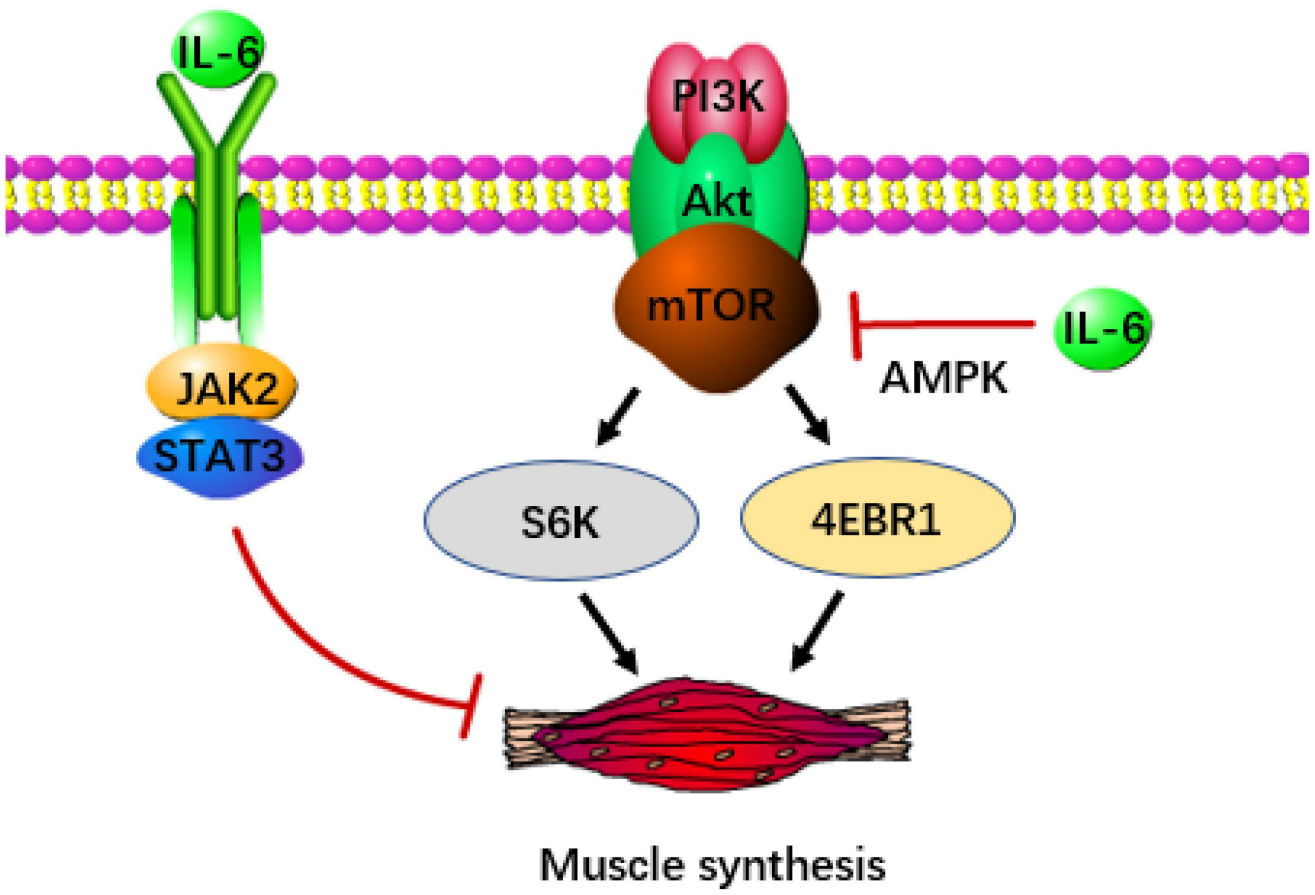
IL-6 accelerates the occurrence of cachexia in pancreatic cancer. IL-6 can inhibit the release of signals from the IGF/Akt/mTOR pathway, thereby reducing the production of S6K and 4EBP-1 (the key transcription factor of muscle synthesis). IL-6 can also directly inhibit muscle synthesis through JAK/STAT3.

Apart from catabolism, the depletion of skeletal muscle also stems from disruptions in anabolism. The IGF/Akt/mTOR signal transduction pathway is crucial for protein and lipid synthesis. Ribosomal S6K and eukaryotic initiation factor 4EBP-1, activated by mTOR, significantly contribute to sustaining muscle growth, making them potentially potent defenses against cachexia (61, 62). Nevertheless, IL-6 induced AMP activated protein kinase (AMPK) showed a negative correlation with mTOR (63), which was detrimental to muscle preservation. In pancreatic cancer, pancreatic stellate cells release significant quantities of IL-6, which signals downstream activation of AMPK. AMPK evidently suppresses mTOR activity (64), ultimately resulting in muscle atrophy. Notably, exercise training could serve as an effective method to suppress AMPK, thereby delaying the advancement of cachexia (65). Additionally, it has been proposed that the IL-6/JAK/STAT3 pathway is a potential contributor to muscle loss, particularly through the phosphorylation of STAT3 (**Figure 2**). Introducing STAT3 into the tibialis anterior of tumor-bearing mice using a plasmid as a vector resulted in noticeable muscle atrophy. This process was accompanied by an increase in the protein degradation gene atrogin-1 (66), although further confirmation is required to establish whether atrogin-1 is associated with the activation of STAT3.

In individuals with lung cancer, Pettersen discovered that the combination of rIL-6R and IL-6 expedited the autophagy of muscle cells through trans-signaling, potentially contributing to muscle loss in patients with cachexia (67). While elevated levels of branched-chain amino acids and protein metabolites are evident in the plasma of pancreatic cancer patients, pointing to autophagy in the advanced stages of pancreatic cancer (68), there is no evidence yet to confirm that this is also triggered by the anti-signal of IL-6. In any event, it can be determined that IL-6 acts as an inducer of cachexia in pancreatic cancer and suppressing IL-6 release represents a target for enhancing prognosis.

## 6 IL-6 and its clinical implications

Using ELISA, it was found that the plasma IL-6 levels in untreated pancreatic cancer patients were significantly higher than those in healthy individuals, and IL-6 level were positively correlated with the tumor stage (8, 69). This finding indicates that IL-6 serves as a valuable diagnostic biomarker for pancreatic cancer. In a study aimed at finding immunobiomarkers, plasma from 73 patients with untreated metastatic pancreatic cancer was tested. Those with high IL-6 expression tended to experience faster metastasis and shorter survival times, highlighting the potential of plasma IL-6 levels in predicting the survival rate of patients with metastatic pancreatic cancer (70).

Considering IL-6’s pivotal role in driving tumorigenesis and metastasis, one might wonder whether inhibiting IL-6 could be a viable strategy in managing the progression of pancreatic cancer. While IL-6 targeted drugs are currently utilized in pancreatic cancer treatment, the effectiveness of individual drugs seems to be limited, likely due to variations among individuals and small sample sizes. Recognizing that tumor development is influenced by multiple factors, it becomes imperative to explore combinations of IL-6 targeted drugs with other medications. Preclinical studies indicate that targeted IL-6 inhibition can enhance the efficacy of anti-PD-L1 therapy in pancreatic cancer (71). Additionally, substances like raloxifene have shown promise in potentiating the effectiveness of chemotherapy agents such as paclitaxel by targeting IL-6 signaling pathways (72). Notably, IL-6 receptor blockade has been shown to improve chemotherapy outcomes in pancreatic cancer. Considering these promising findings, there is strong rationale to believe that IL-6 antibodies hold significant potential in combination therapies for pancreatic cancer (73).

## 7 Summary

After the discovery of IL-6, its role in promoting the growth, invasion, and spread of cancer has been widely recognized. The use of IL-6 monoclonal antibody has shown promising results in treating renal cell carcinoma, prostate cancer, lymphoma, multiple myeloma, and other diseases (74–76). However, clinical trials for pancreatic cancer treatment are still pending. It’s important to note that due to the short half-life of the IL-6 antibody, repeated administration is necessary to maintain effective blood concentration (77). Research into the development of IL-6 receptor antibodies could potentially improve therapeutic effectiveness and enhance patient convenience (78).

## Author contributions

MS: Conceptualization, Funding acquisition, Project administration, Writing – original draft. YT: Validation, Investigation, Writing – original draft. KC: Writing – original draft, Data curation. KX: Supervision, Visualization, Writing – review & editing. LQ: Writing – review & editing, Conceptualization, Project administration, Validation.
